# Case Report: Single-cell RNA sequencing reveals cellular and molecular mechanisms in newborn cardiac hemangioma formation

**DOI:** 10.3389/fcvm.2025.1682677

**Published:** 2025-12-08

**Authors:** Tiange Li, Qi An, Shuhua Luo, Yifei Li

**Affiliations:** 1 Key Laboratory of Birth Defects and Related Diseases of Women and Children of MOE, Department of Pediatrics, West China Second University Hospital, Sichuan University, Chengdu, China; 2 Department of Cardiovascular Surgery, West China Hospital, Sichuan University, Chengdu, China

**Keywords:** single-cell RNA sequencing, cardiac hemangioma, neonate, molecular mechanism, VEGF signals

## Abstract

Primary cardiac hemangiomas are extremely rare benign tumors, with limited molecular characterization available. This study investigated a case of mixed-type intracardiac hemangioma in a 17-day-old female neonate, initially detected via prenatal echocardiography and confirmed by postoperative histopathology. The right atrial mass (1.7 × 2.0 × 2.1 cm) was surgically resected, and 1-year follow-up, which included transthoracic echocardiography every 3 months and cardiac MRI every 6 months, showed no recurrence and normal cardiac function. Single-cell RNA sequencing was performed on the tumor tissue, yielding 4,888 high-quality cells after quality control. These cells were classified into 9 distinct types, with fibroblasts/myofibroblasts and smooth muscle cells accounting for nearly half the population. Endothelial cells were subdivided into two clusters: Cluster 1, enriched in immune inflammation, cell adhesion, and signal transduction. And Cluster 2, focused on mitochondrial energy metabolism and ribosome biogenesis. InferCNV analysis revealed relative genomic stability, with only minor copy number variations on chromosome 13 in Cluster 1, supporting the tumor's benign nature. Cell communication analysis identified Cluster 1 as the primary effector cell in hemangioma formation, receiving VEGF signals mainly from myeloid-derived suppressor cells and common myeloid progenitors, while also driving collagen synthesis-related pathways. This study provides critical insights into the cellular and molecular mechanisms of cardiac hemangiomas, filling gaps in current understanding of this rare tumor.

## Introduction

Primary cardiac tumors constitute an exceptionally rare pathological entity, with autopsy-based studies documenting an incidence of 0.0017%–0.27% (1). Among these lesions, benign neoplasms account for 75% of all primary cardiac tumors, with myxomas alone comprising 50% of these benign cases, whereas hemangiomas only constitute 2.8% (1). Cardiac hemangiomas are characterized by benign endothelial cell proliferation and share identical histological features with their extracardiac counterparts. These lesions may arise from any cardiac structure, including the atria, ventricles, interventricular septum, epicardial surface, and pericardium (2, 3). Clinical detection of cardiac hemangiomas remains challenging, as symptoms typically manifest only when the tumor reaches a sufficient size or occupies a strategically critical anatomical location (4, 5). Key presenting symptoms include dyspnea, palpitations, chest pain, and cardiac arrhythmias (2). Surgical resection remains the first-line treatment, with generally favorable outcomes; however, long-term recurrence rates remain inadequately characterized.

Histologically, hemangiomas are categorized into three distinct subtypes, capillary (characterized by small-caliber vessels analogous to capillaries), cavernous (featuring multiple dilated, thin-walled vascular spaces), and mixed-type (exhibiting combined features of the aforementioned two subtypes) (6). This classification framework is equally applicable to cardiac hemangiomas. Capillary hemangiomas typically present in infancy, undergoing an initial proliferative phase characterized by rapid growth, which is subsequently followed by spontaneous involution with advancing age (7). Notably, the proliferative phase is marked by the upregulation of vascular endothelial growth factor A (VEGFA) and fibroblast growth factor receptor 1 (FGFR1) (8, 9). Extensive research efforts have focused on cavernous hemangiomas, which are clinically subclassified into hepatic, retinal, and cerebral variants (10). Emerging evidence from recent investigations indicates that mutations in specific genes—including *KRIT1, CCM2, PDCD10, and MAP3K3*—may drive the pathogenesis of cavernous hemangiomas (11–13).

In cardiovascular research, studies on inflammatory regulation, molecular biomarkers and prognostic models, and single-cell analysis inform single-cell sequencing of cardiovascular tumors for pathological and precision medicine exploration (14–17). Single-cell RNA sequencing (scRNA-seq) has emerged as a robust tool for dissecting tumor biology, providing unprecedented insights into cellular heterogeneity, gene expression profiles, immune microenvironment composition, and intercellular communication networks (18–20). Recent studies have leveraged scRNA-seq to investigate the pathogenesis of vascular hemangiomas and identify critical cellular populations (10–13). Nevertheless, a comprehensive molecular characterization of primary cardiac hemangiomas remains lacking in the current literature. In the present study, we report a rare case of mixed-type cardiac hemangioma in a neonate and employ scRNA-seq to delineate the transcriptional landscape across distinct cell populations, thereby elucidating the molecular mechanisms underlying cardiac hemangioma formation.

## Methods and materials

### Ethics approval and consent to participate

The study protocol was approved by the Ethics Committee of the Second West China Hospital of Sichuan University (Approval No. 2019-061). Written informed consent was obtained from the patient's guardian prior to sample collection. The cardiac hemangioma sample was surgically resected from a 17-day-old female neonate, and all procedures adhered to the Declaration of Helsinki.

### Sample processing and single-cell RNA sequencing

Tumor tissue was dissociated into single-cell suspensions using the Tumor Dissociation Kit (Miltenyi Biotec, Catalog No. 130-095-929), combining mechanical dissociation (gentleMACS™ Dissociators, Miltenyi Biotec) and enzymatic digestion to preserve cellular integrity. The suspension was filtered to remove debris (>10,000 bp) before downstream processing. Single-cell libraries were prepared using the Chromium™ System (10x Genomics) with the Chromium Next GEM Single Cell 3ʹ Library Kit v3.1 (Catalog No. PN-1,000,157). Cells were adjusted to 700–1,200 cells/µL (viability ≥85%), and ∼8,000 cells were loaded per lane to generate bead-in-emulsions (GEMs). After reverse transcription, barcoded cDNA was purified (Dynabeads, Thermo Fisher Scientific) and amplified by PCR. The 3ʹ gene expression library was constructed via cDNA fragmentation, end-repair, and double-size selection (SPRIselect beads, Beckman Coulter), then sequenced on an Illumina NovaSeq platform (150 bp paired-end reads).

### RNA-Seq data processing and quality control

Raw data were processed with the Cell Ranger pipeline (10x Genomics) to generate a UMI count matrix. All downstream analyses were performed in R (v4.5.1) using the Seurat package (v5.3.0). Cells were retained if they met the following quality control (QC) criteria: detected genes ranking from 200 to 4,000, mitochondrial gene percentage <5% and minimum 3 cells expressing each gene. After QC, 4,888 high-quality cells were retained from the initial 8,668 cells (Supplementary Figures S1A–D).

### Data normalization, dimensionality reduction, and cell clustering

The UMI matrix was normalized using LogNormalize (scale factor = 10,000) to calculate log2(TPM + 1) values. Scaling was performed with ScaleData to adjust for confounding variables. The top 2,000 highly variable genes (HVGs) were identified via variance-stabilizing transformation (vst method) using FindVariableFeatures (implemented in Seurat v5.3.0). Principal Component Analysis (PCA) was performed on HVGs, and the top 15 PCA dimensions were selected based on an elbow plot. Cells were clustered using FindNeighbors (top 15 PCA dimensions) and FindClusters (resolution = 0.5). Uniform Manifold Approximation and Projection (UMAP) was applied to the top 15 PCA dimensions for visualization (Supplementary Figures S1E–H).

### Differential expression analysis

Differential expression analysis was performed using Seurat::FindAllMarkers (v5.3.0) with the following parameters: only.pos = TRUE, min.pct = 0.25, logfc.threshold = 0.25, Statistical test: Wilcoxon rank-sum test.

### Cell type annotation

Cell types were annotated using a combination of reference-based and marker-guided approaches. Reference-based annotation: The SingleR package (v2.10.0) was used to annotate clusters against the HumanPrimaryCellAtlasData reference (celldex), generating broad and fine-grained cell type labels. Marker validation: Differential markers per cluster were identified via Seurat::FindAllMarkers (Wilcoxon rank-sum test; only.pos = TRUE, min.pct = 0.25, logfc.threshold = 0.25). Canonical markers were validated using FeaturePlot and DotPlot (implemented in Seurat v5.3.0). Final cell types included smooth muscle cells, fibroblasts/myofibroblasts, monocytes/macrophages, two endothelial cell clusters, T cells, natural killer (NK) cells, B cells, and common myeloid progenitors.

### Endothelial cell subset analysis

The two endothelial cell clusters were isolated for focused analysis. The subset was re-normalized, scaled, and re-clustered (via Seurat v5.3.0) to confirm subpopulation separation. Differential genes between the two clusters were identified via Seurat::FindMarkers (ident.1 = “Endothelial cell cluster1”, ident.2 = “Endothelial cell cluster2”, min.pct = 0.1, logfc.threshold = 0.25), and results were exported for downstream enrichment analysis.

### Gene enrichment analysis

Functional enrichment analysis of differential genes was performed using the clusterProfiler ecosystem (including enrichplot v1.28.4, DOSE v4.2.0, GOSemSim v2.34.0, and fgsea v1.34.2) and org.Hs.eg.db (human gene annotation database), with a false discovery rate (FDR) cutoff of <0.05. Gene Ontology (GO) enrichment (Biological Process, Molecular Function, Cellular Component) was conducted after converting gene symbols to Entrez IDs via bitr. Key results were visualized using dotplot, emapplot (GO term network), and cnetplot (gene-term network).

### InferCNV analysis

Copy number variation (CNV) analysis was performed using infercnv (v1.24.0) to assess genomic stability. Input data Filtered UMI matrix and gene chromosomal positions (from TxDb.Hsapiens.UCSC.hg38.knownGene, integrated with GenomeInfoDb v1.44.3); Reference group was “Endothelial cell cluster2” (designated as the normal reference). Other parameters: cutoff = 0.1 (optimal for 10x data), denoise = TRUE, HMM = TRUE (hidden Markov model for CNV prediction), out_dir = “infercnv_output”.

### Cell communication analysis

Cell-cell communication was analyzed using CellChat (v1.6.1) with the CellChatDB.human signaling database. CellChat predicts the probability of cell-cell communication by combining gene expression data with established knowledge of interactions between signaling ligands, receptors, and their cognates, utilizing a comprehensive set of interaction rules. Overexpressed genes and ligand-receptor pairs were identified using identifyOverExpressedGenes and identifyOverExpressedInteractions. Communication probabilities were calculated via computeCommunProb, and interactions with ≥10 supporting cells were retained (filterCommunication).

### Statistical and software details

Key software packages and versions (consistent with sessionInfo() output) were shown as Supplementary Materials.

## Results

### Case presentation and hemangioma sample collection

A 17-day-old female neonate was referred to our institution for evaluation of a cardiac mass that was initially identified during routine prenatal echocardiographic screening. Fetal echocardiography revealed a right atrial mass measuring 1.3 × 0.9 cm, characterized by cystic echogenicity with thickened walls and an absence of intracavitary flow signals on Doppler interrogation. Following multidisciplinary consultation, it was recommended that the pregnancy be continued with serial fetal echocardiographic monitoring to assess tumor growth and evaluate cardiovascular function.

**Figure 1:**
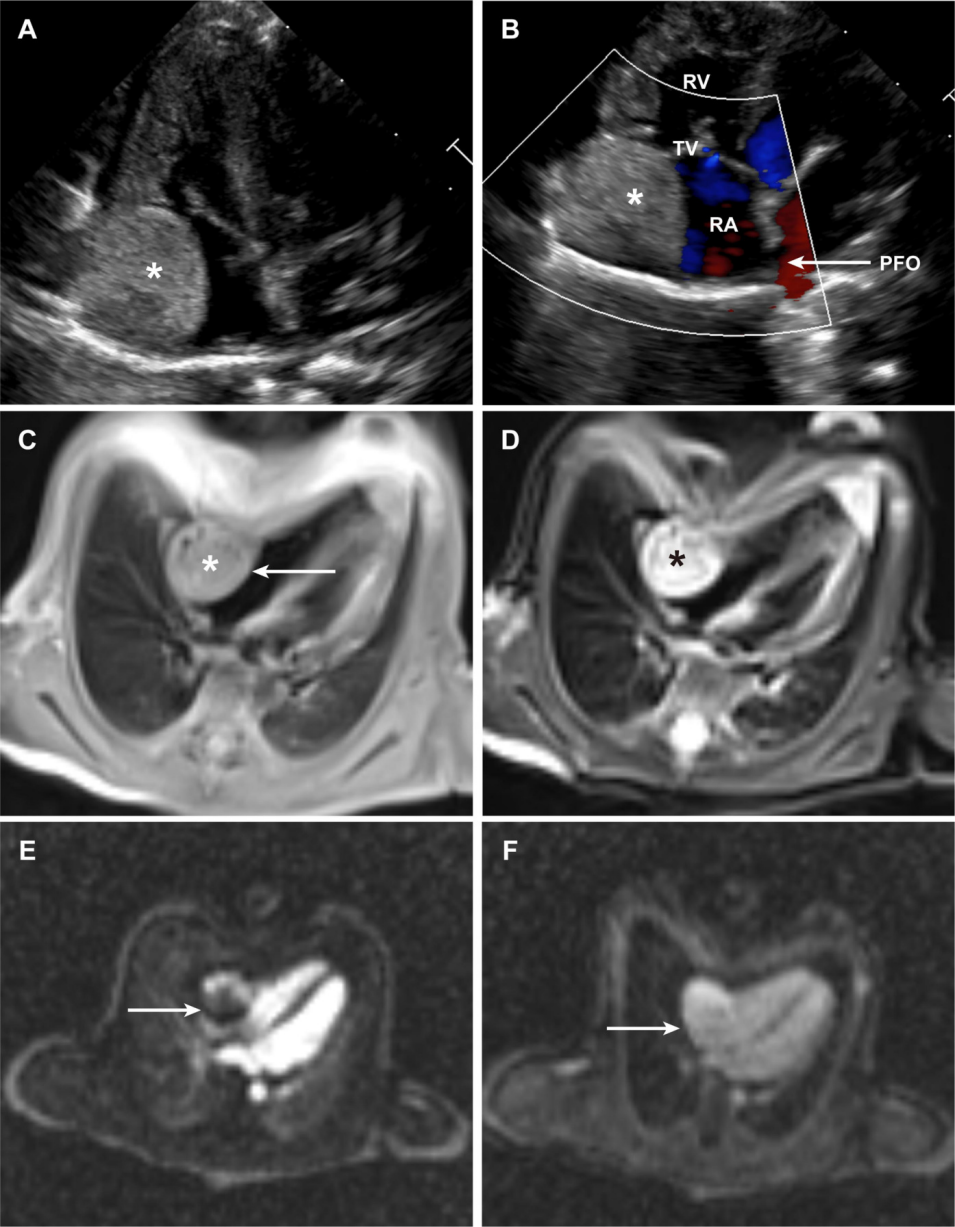
Clinical images of the patient. **(A)** Transthoracic echocardiographic image of the intracardiac hemangioma. **(B)** Color Doppler image demonstrating intracardiac blood flow. **(C,D)**, Cardiac MRI image of the intracardiac hemangioma in the T1-weighted **(C)** & T2-weighted **(D)** sequence. **(E)** Cardiac MRI image of the intracardiac hemangioma from first-pass perfusion imaging. **(F)** Cardiac MRI image of the intracardiac hemangioma from delayed enhancement imaging. (PFO, patent foramen ovale; RA, right atrium; RV, right ventricle; TV, tricuspid valve).

The patient was delivered at term and admitted to our cardiac intensive care unit on the eighth postnatal day. At presentation, vital signs were stable, as follows: temperature 36.9 °C, heart rate 144 beats per minute, respiratory rate 45 breaths per minute, and blood pressure 75/45 mmHg. Physical examination revealed no dysmorphic features or signs of congestive heart failure. Transthoracic echocardiography showed normal cardiac chamber dimensions and preserved biventricular function. No signs of heart failure were observed, with left ventricular ejection fraction (LVEF) of 60% and fractional shortening (FS) 31%. A well-demarcated, slightly hyperechoic mass measuring 1.7 × 2.0 × 2.1 cm was identified along the lateral wall of the right atrium, which exhibited regular morphology with distinct borders (Figure 1A). Additional findings included a patent foramen ovale with left-to-right shunting and mild tricuspid regurgitation (Figure 1B). Cardiac magnetic resonance imaging was performed for comprehensive tissue characterization: T1-weighted sequences revealed isointense signal intensity relative to the myocardium (Figure 1C), whereas T2-weighted fat-suppressed images demonstrated hyperintense signal intensity (Figure 1D); cine imaging showed indistinct delineation between the mass and the adjacent right atrial wall; first-pass perfusion imaging revealed heterogeneous contrast uptake, with prominent peripheral enhancement and central hypoperfusion (Figure 1E); late gadolinium enhancement sequences demonstrated significant contrast retention within the lesion (Figure 1F).

Given the imaging features and potential for growth-associated complications, surgical resection was recommended. The procedure was performed via median sternotomy under cardiopulmonary bypass, with bicaval cannulation and aortic cross-clamping. Cardioplegic arrest was achieved, followed by performance of a transverse right atriotomy. Intraoperative inspection revealed a pale, firm mass with a “fish-flesh” appearance. The tumor showed intimate adherence to the endocardial surface, without transmural extension or invasion of the atrial wall. Complete tumor excision was achieved with negative margins, and the integrity of the right atrial wall was preserved. Following meticulous hemostasis and closure of the atriotomy, the patient was successfully weaned from cardiopulmonary bypass, with stable hemodynamics. Histopathological examination confirmed a mixed-type cardiac hemangioma, containing both capillary and cavernous components: the capillary regions showed proliferation of small-caliber vessels lined by benign endothelial cells, whereas the cavernous regions exhibited dilated, thin-walled vascular spaces. No evidence of malignancy, mitotic activity, or cellular atypia was observed. Thus, the diagnosis of “intracardiac mixed-type hemangioma” was clearly confirmed in this neonate. During the one-year postoperative follow-up, the neonate underwent transthoracic echocardiography every three months and cardiac magnetic resonance imaging every six months. All assessments confirmed that cardiac function parameters remained within the normal reference range (LVEF: 58%–62% and FS: 30%–33%), with no arrhythmia, chamber dilatation, or tumor recurrence. When the neonate reached one year of age, she weighed 8.5 kg and had a body length of 70 cm.

### Identification of cellular and molecular landscape of cardiac hemangioma

scRNA-seq was performed on the resected cardiac hemangioma tissue, generating transcriptomic profiles of 8,668 individual cells. To guarantee high-quality data for subsequent analysis, stringent filtering criteria were implemented to exclude cellular debris, potential doublets, and apoptotic cells. Following the implementation of these quality control procedures, 4,888 high-quality cells were retained for downstream analytical processes. Based on elbow plots, clustree analysis, and UMAP plots under different resolutions, a resolution of 0.5 was determined for downstream analysis. Ultimately, 15 cell clusters were obtained (Supplementary Figure S1).

**Figure 2:**
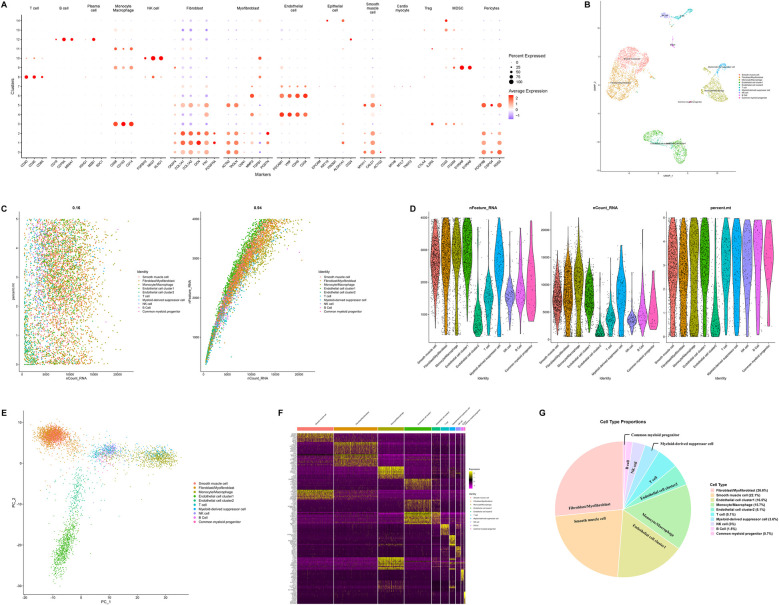
Integrated single-cell RNA sequencing analysis revealing cellular heterogeneity, cell type identification, and molecular features. **(A)** Dot plot illustrating expression patterns of marker genes across distinct cell clusters. **(B)** Uniform manifold approximation and projection plot visualizing cellular heterogeneity. Cells are colored by cell types to illustrate the diversity of cell populations in the sample. **(C)** Scatter plots (left) showing the total number of transcripts vs. the percentage of mitochondrial genes for each cell type, and the right plot verifying internal data consistency. **(D)** Violin plots displaying the number of detected genes, total transcripts, and mitochondrial gene percentage across different cell types. The data has undergone quality control and standardization. **(E)** Principal component analysis plot showing cell distribution in the principal component space, with cells colored by cell type. **(F)** Heatmap illustrating expression profiles of differentially expressed genes across cell types. **(G)** Pie chart showing the proportion of each cell type in the sample. (PCA, principal component analysis; QC, quality control; scRNA-seq, single-cell RNA sequencing; UMAP, uniform manifold approximation and projection).

For cell type annotation, both manual and automated annotation were employed to ensure the reliability of the results. Dot plots visualized the manual annotation of cell types. In this plot, the lower part displays highly expressed specific markers for various cell types, while the upper part shows the corresponding cell types. The *y*-axis represents the serial numbers of cell clusters (Figure 2A). Ultimately, all cells were classified into 9 cell types, namely smooth muscle cell, fibroblast/myofibroblast, monocyte/macrophage, endothelial cell, T cell, NK cell, B cell, myeloid-derived suppressor cell, and common myeloid progenitor (Figure 2B). Among these, fibroblast/myofibroblast and smooth muscle cell accounted for nearly half of the total cell count, followed by endothelial cell and other immune cell (Figure 2G). Figures 2C–E illustrates the quality control metrics and principal component visualizations with cell type annotations. Finally, a heatmap was used to display the specifically highly expressed markers of different cell clusters (Figure 2F).

**Figure 3:**
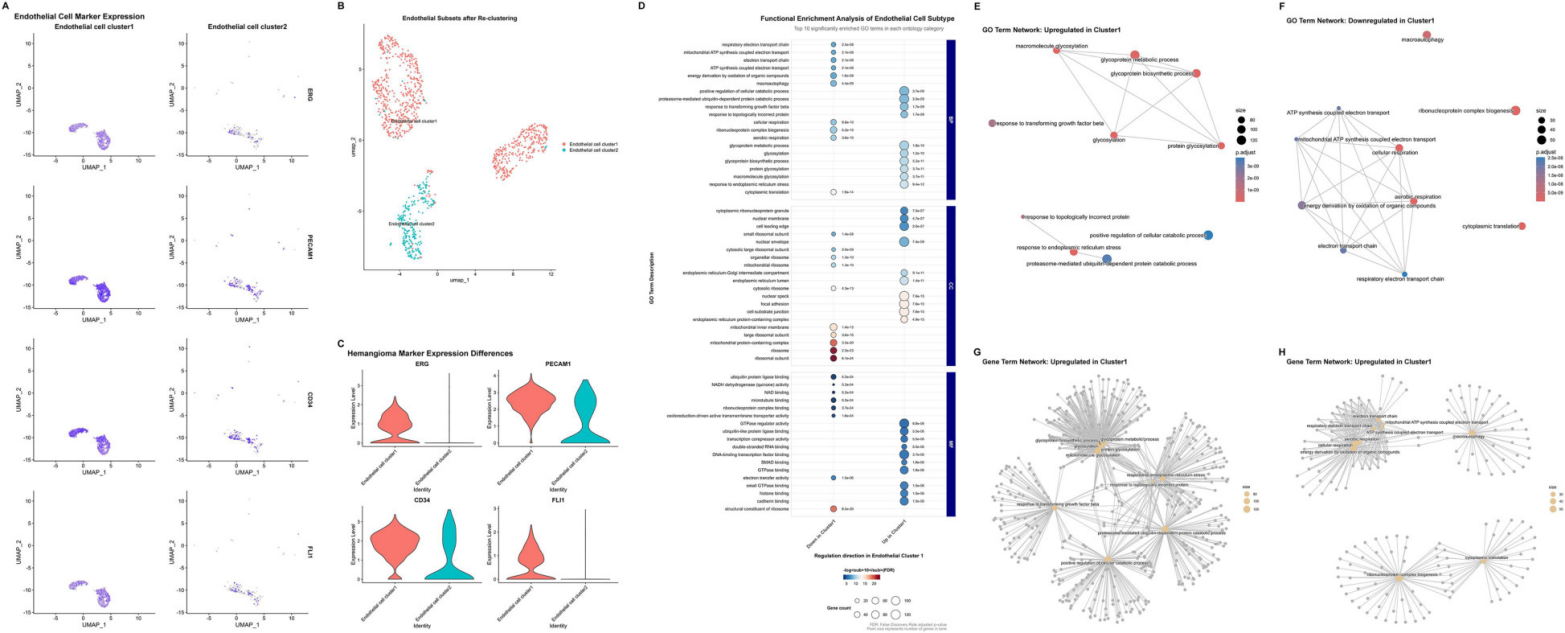
Single-cell transcriptomic profiling of endothelial subtypes and hemangioma-related molecular features. **(A)** Uniform manifold approximation and projection plots illustrating the different expression marker genes (*ERG*, *PECAM1*, *CD34*, *FLI1*) of hemangioma in endothelial cells subtypes. **(B)** Uniform manifold approximation and projection plots depicting the re-clustering results after extracting endothelial cells. **(C)** Violin plots showing the expression differences of hemangioma marker genes (*ERG*, *PECAM1*, *CD34*, *FLI1*) across distinct endothelial cell types. **(D)** Functional enrichment analysis of endothelial cell subtypes, presenting the top 10 significantly enriched gene ontology terms across each category (Biological Process, Cellular Component, and Molecular Function). **(E,F)** Upregulated **(E)** and downregulated **(F)** gene ontology term network in Endothelial cell cluster 1. **(G,H)** Upregulated **(G)** and downregulated **(H)** gene term network Endothelial cell cluster 2. (UMAP, uniform manifold approximation and projection; GO, Gene Ontology.).

Postoperative tumor tissues were analyzed using hematoxylin-eosin (HE) staining and immunohistochemistry (IHC). Mitotic activity was quantified as the mitotic index (number of mitotic cells per 10 high-power fields less than 1). Malignancy-related markers were evaluated by IHC, with the following results: Ki67 positivity rate less than 2%, negative Aurkb expression, and no abnormal expression of malignant markers (such as p53, CK7). IHC of postoperative pathological sections confirmed high expression of *ERG*, *FLI1*, *CD34* and *PECAM1* in tumor cells. Based on this finding, the endothelial cell cluster was divided into two subpopulations, designated as Endothelial cell cluster 1 (“Cluster 1” in following description) and Endothelial cell cluster 2 (“Cluster 2” in following description) (Figures 3A–C). UMAP enabled clear distinction between the two endothelial cell subpopulations (Figure 3B). These two cell subpopulations were isolated separately and subjected to re-comparative analysis to identify highly variable features between them. Enrichment analysis revealed significantly differential Gene Ontology (GO) terms between the two subpopulations (Figure 3D). Endothelial cells constitute a highly heterogeneous population exhibiting diverse morphological, phenotypic, and functional characteristics (21, 22). Previous studies have established the central role of endothelial dysfunction in hemangioma pathogenesis (13). In terms of biological process (BP), Cluster 1 is predominantly enriched in glycosylation-related processes, protein catabolic processes, and stress responses, whereas Cluster 2 is significantly enriched in mitochondrial energy metabolism and ribosome biogenesis. For cellular component (CC), Cluster 1 is highly enriched in cell adhesion structures, while Cluster 2 is highly enriched in mitochondrial and ribosomal components. Regarding molecular function (MF), Cluster 1 is remarkably enriched in signal transduction-related binding activities and transcriptional regulatory activities, and Cluster 2 is significantly enriched in energy metabolism-related activities. Collectively, Cluster 1 tends to be involved in immune inflammation, cell adhesion, and signal transduction; in contrast, Cluster 2 focuses on mitochondrial energy metabolism, ribosome biogenesis, and maintenance of basic cellular components. The functional differences between the two reflect the subpopulation specialization of endothelial cells in cardiac hemangiomas. GO term network analysis and gene-term network analysis also confirmed this significant difference (Figures 3E–H, Supplementary Figure S2).

### InferCNV analysis uncover the degree of malignancy in cells

Given that somatic mutations are fundamental drivers of tumorigenesis, we sought to assess the presence of malignant transformation in these cell populations and elucidate potential oncogenic mechanisms. Single-cell transcriptomic data enabled the identification of key genomic features through multi-step computational analysis, including the estimation of copy number variations (CNVs) across large chromosomal segments in individual cells using the inferCNV algorithm (Supplementary Figure S3). With Cluster 2 serving as control cells, analysis of other cell populations revealed that chromosomal copy number alterations in somatic cells were primarily concentrated on chromosome 1, most prominently in smooth muscle cells and fibroblasts/myofibroblasts. For immune cells, chromosomal copy number alterations were mainly focused on chromosomes 9 and 10, with the most significant changes observed in monocytes/macrophages. These apparent CNVs likely reflect the high expression levels of chromosome-resident genes characteristic of these cell lineages, rather than representing true genomic instability.

Notably, compared with Cluster 2, endothelial cell Cluster 1 exhibited a slight increase in CNVs on chromosome 13, while no significant copy number alterations were detected across other chromosomes. This relative genomic stability within the vascular compartment is consistent with findings from established literature, which demonstrates that capillary and cavernous hemangiomas lack malignant potential and do not harbor recurrent chromosomal aberrations. These results suggest that the pathogenesis of hemangiomas involves epigenetic dysregulation or developmental anomalies, rather than classical oncogenic mutations, supporting their classification as benign vascular proliferations rather than true neoplasms.

### Cell-cell communication analysis reveals the process of vascular hemangioma formation

**Figure 4:**
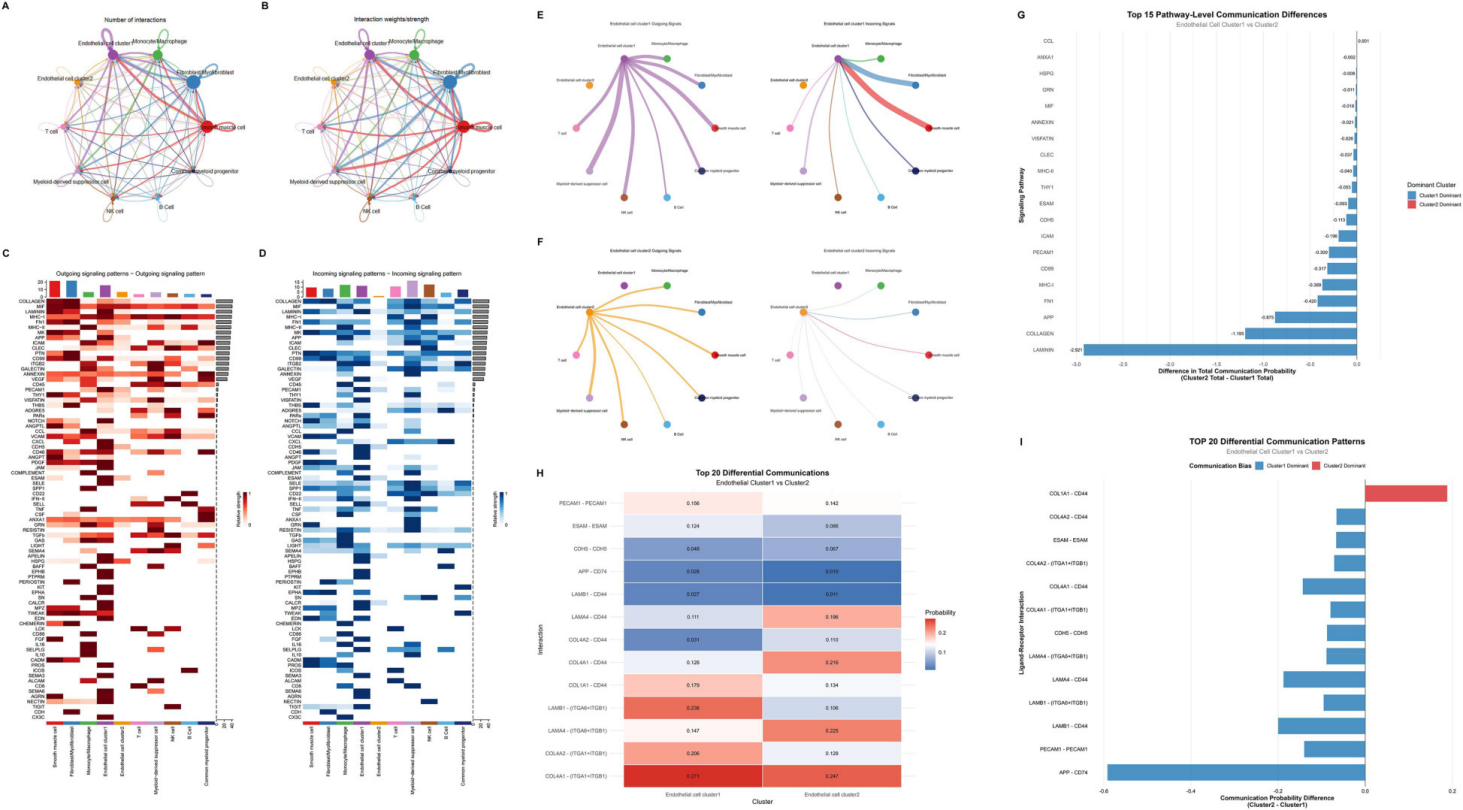
Cell communication analysis between endothelial cell clusters. **(A,B)** Cell interaction networks showing the number of interactions **(A)** and interaction weights **(B)** among different cell types. **(C,D)** Heatmaps depicting outgoing **(C)** and incoming **(D)** signaling patterns across various signaling pathways. **(E,F)** Diagrams illustrating outgoing (left) and incoming (right) signals of Endothelial cell cluster 1 **(E)** and cluster 2 **(F) (G)** Bar plot of the top 15 pathway-level communication differences between Endothelial cell cluster 1 and cluster 2, indicating the difference in total communication probability and dominant cluster for each pathway. **(H)** Heatmap of the top 20 differential ligand-receptor mediated communications between Endothelial cell cluster 1 and cluster 2, showing the communication probabilities for each ligand-receptor interaction. **(I)** Bar plot of the TOP 20 differential ligand-receptor mediated communication patterns, displaying the communication probability differences and biases between Endothelial cell cluster 1 and cluster 2 for each ligand-receptor interaction.

To investigate intercellular communication networks within the tissue microenvironment, we employed CellChat analysis to quantify cell-cell interactions among the 10 identified cell clusters (Figures 4A,B). In terms of the quantity and intensity of cell-cell interactions, somatic cells were significantly higher than those of immune cells. Meanwhile, the outgoing and incoming signaling pathways of the 10 cell clusters were also visualized (Figures 4C,D). Smooth muscle cell, fibroblast/myofibroblast, and endothelial cell Cluster 1 are the cell types that emit the most signaling pathways and also receive a relatively large number of signaling pathways. Among immune cells, myeloid-derived suppressor cell and monocyte/macrophage are the cell types that receive the most signals. Meanwhile, compared with Cluster 2, Cluster 1 receives more angiogenesis- and inflammation-related signaling pathways, such as those associated with VEGF and PECAM1.

Separate analysis was conducted on the cell communication differences between the two endothelial cell populations. In terms of the quantity of cell communication, Cluster 1 was significantly stronger than Cluster 2 (Figures 4E,F). Detailed analysis of Cluster 1 revealed that this cell population exhibited the closest intercellular communication with fibroblasts/myofibroblasts and smooth muscle cells. Among immune cells, monocytes/macrophages and myeloid-derived suppressor cells showed the most intimate cell communication with Cluster 1. Comparative analysis of the cell communication pathways and ligand-receptor interactions between Cluster 1 and Cluster 2 enabled the extraction of top-ranked differential pathways and ligand-receptor interactions (Figures 4G–I, Supplementary Figure S4). It was found that Cluster 1 ranked highly in fibrosis and gliogenesis pathways (such as LAMININ, COLLAGEN, and FN1 pathways) as well as in ligand-receptor interactions. This suggests that the mechanism underlying hemangioma formation may be associated with inflammation and fibrosis.

## Discussion

Hemangiomas represent the most common benign vascular tumors of infancy, with recent epidemiological studies reporting an overall incidence of 2%–10%, demonstrating a marked female predominance (23). These lesions can manifest at any anatomical location, with preferential involvement of the head and neck region (24, 25). Based on vascular architecture, hemangiomas are classified into capillary, cavernous, and mixed subtypes according to the caliber of affected vessels (6). Capillary hemangiomas, colloquially termed “strawberry hemangiomas,” predominantly affect infants and typically present as superficial, erythematous, raised lesions (7). The natural history of capillary hemangiomas follows a characteristic biphasic progression encompassing proliferative and involutional phases. The proliferative phase occurs during the initial 3–5 months of life, characterized by rapid endothelial cell proliferation and accelerated tumor growth (24, 26, 27). This phase typically culminates by 9–12 months of age (24, 26). Subsequently, the involutional phase commences around 12 months and may persist for 3–9 years (28). During involution, endothelial cells undergo progressive replacement by fibrofatty tissue (24), resulting in residual anatomical changes in approximately 69% of patients (29). In contrast, cavernous hemangiomas are characterized by deeper dermal involvement and characteristic blue discoloration, typically present at birth. These lesions demonstrate proportionate growth with somatic development and lack spontaneous regression potential (7). Cavernous hemangiomas are more accurately classified as vascular malformations, representing developmental anomalies involving abnormal capillary or small arterial vessel dilatation (30).

However, extracutaneous involvement remains uncommon, with hepatic manifestations representing the predominant extracutaneous site, followed by gastrointestinal, cerebral, mediastinal, and pulmonary locations (31). The heart is uncommon affected by hemangiomas, accounting for only 2.8% of primary tumors of the heart (1). Cardiac hemangiomas can arise throughout the heart, with the right atrium being the most frequently affected chamber (32). Rare localizations, including the atrioventricular node and coronary sinus, have been documented (33, 34). Histologically, cardiac hemangiomas often demonstrate mixed architectural features of both capillary and cavernous subtypes (35). Patients with cardiac hemangiomas typically remain asymptomatic until hemodynamic compromise or local invasion occurs. Exercise intolerance represents the most common presenting symptom, while serious cardiovascular events including syncope, angina, and stroke occur in approximately 20% of cases (35). Hemangiomas involving the cardiac conduction system may precipitate atrioventricular block or sudden cardiac death (36, 37). Although cardiac hemangiomas generally demonstrate a benign histopathological presentation, but cardiac hemangiomas still had been identified with significant clinical risks that necessitate prompt evaluation and management. Imaging examination is of great value in preoperative screening and diagnosis of cardiac hemangiomas. Echocardiography allows real-time observation of blood flow signals within the mass, providing significant evidences in diagnosing hemangiomas (32). Cardiac magnetic resonance imaging can reveal the natural structure of soft tissue masses and is also important in cardiac hemangiomas diagnosis. Typically, cardiac hemangiomas exhibit intermediate to high signal intensity on T1-weighted and T2-weighted images, respectively, and demonstrate typical rapid, uniform enhancement after contrast injection (38). With advancements in imaging technology, an increasing number of cardiac hemangiomas are being detected. However, due to the rarity, clinical understanding of this condition usually relies on case reports or literature review, lacking standardized clinical guidelines for diagnosis and management. Therefore, the surgical indication for cardiac hemangiomas remains controversial. For symptomatic patients, surgical resection is commonly employed in clinical practice, although some experts advocate for pharmacological treatment.

Endothelial cells have been characterized by significant heterogeneities in both structure and function among different tissues (39). Endothelial cells have been identified to be involved in various crucial roles in the vascular system, including vascular wall formation, regulation of immune responses, modulation of vascular dynamics, and participation in blood coagulation (39–41). This heterogeneity arises from the ability of endothelial cells to sense changes in their microenvironment and exhibit different cell phenotypes, resembling a nonlinear input/output device (42). Therefore, endothelial cells exhibit significant heterogeneity at different locations or times. Additionally, the phenotype of endothelial cells is regulated by epigenetics, with endothelial cells at specific locations exhibiting certain fixed phenotypes (40). Hemangiomas are tumors formed by excessive proliferation of vascular endothelial cells, mediated by vascular endothelial growth factor (VEGF) signaling through VEGFR2 (43). In the process of hemangioma formation, vessel growth and expansion are key mechanisms, and the primary driving factor is VEGF. It is generally believed that VEGF is released by hypoxic tissues and binds to VEGFR2 expressed on endothelial cells, promoting endothelial cell proliferation and migration. Endothelial cells with high levels of VEGFR2 signaling pathway become tip cells and induce neighboring endothelial cells to become stalk cells by upregulating the Notch ligand Delta-like 4 (DLL4). Then DLL4 binds to NOTCH1 receptors expressed on stalk cells, suppressing the VEGFR2 pathway and inducing the VEGFR1 pathway, thereby enhancing the stalk cell behavior of endothelial cells. This process is the sprouting mechanism of vascular growth (44), both normal vascular growth and vascular growth in tumors primarily occur through this pathway. There are currently numerous studies investigating the role of the VEGF/VEGFR2 signaling pathway in the development of hemangiomas. Zhang et al. recently revealed hypoxia-inducible factor 1-alpha (HIF-1*α*) regulates the cell cycle of vascular endothelial cells through the VEGF/VEGFR2 pathway, thereby stimulating their proliferation, inhibiting apoptosis, and promoting the formation of hemangiomas (45). Studies have also identified several downstream cellular factors in this pathway, including ERK1/2, STAT3, and AKT (46–48). The VEGF/VEGFR2 signaling pathway promotes endothelial cell growth and hemangioma formation by activating these cellular factors. In the cell communication analysis, Cluster 1 demonstrated high-level regulation of the VEGF signaling pathway and received VEGF signals emitted by nearly all other cell populations. By contrast, Cluster 2 exhibited almost no reception of regulatory signals from the VEGF signaling pathway. This indicates that Cluster 1 is the primary effector cell in hemangioma formation. In summary, our single-cell sequencing analysis investigated the significant upregulation of the VEGF pathway in endothelial cells of cardiac hemangiomas, promoting the formation of the tumor.

Some previous studies have also performed single-cell sequencing on hemangiomas and explored the possible mechanisms underlying their occurrence and development. Orsenigo et al. focused on cerebral cavernous malformations (CCM) and identified endothelial cells as core lesion-related cells, similar to our finding that endothelial cells drive cardiac hemangioma formation (13). The study also confirmed critical role of VEGF pathway in vascular lesion progression. Additionally, they reported arterial endothelial cells are resistant to CCM transformation, whereas we split cardiac ECs into two functional clusters. One involved in immune inflammation and cell adhesion as the main lesion driver, and the other focused on mitochondrial metabolism. Ji et al. studied cavernous hemangiomas and emphasized such lesions' benign nature via genomic stability analysis, consistent with our InferCNV results showing only minor chromosomal variations in endothelial cells (10). But they proposed embryonic mesenchymal stem cells as the lesion origin, while in our sequencing data, the proportion of immune cells falls within the normal range. Immune-related signals in cell-cell communication may be associated with vascular homeostasis maintenance rather than tumor microenvironment remodeling. This finding further confirms the characteristics of hemangioma as a benign tumor. The study also linked ECs to collagen synthesis pathways that may relate to hemangioma involution, a point not addressed in their work.

Laminin, FN1 and collagen are widely present in the extracellular matrix of the normal heart, serving as crucial components of the extracellular framework (49). Under the induction of various pathological factors, activation of diverse inflammatory pathways leads to a significant upregulation of fibrosis related cytokines and mediator's expression, resulting in extracellular matrix deposition and fibrosis aggressive formation (49, 50). Currently, there are limited researches focusing on the role of fibrosis in the progression of hemangiomas. During the involution phase of hemangiomas, the proliferation of endothelial cells terminated and gradually replaced by fibrofatty tissue. In the separately extracted cell communication analysis, it can be observed that Cluster 1 emits significantly more collagen synthesis-related signaling pathways compared with Cluster 2 (Figures 4G–I). Thus, we hypothesized that laminin, FN1 and collagen may play crucial roles in this process. Adult hemangiomas trend to form a complete fibrous capsule, and extracellular matrix components are key to its formation (10, 13). While pediatric hemangiomas are mainly composed of fibrous septa, collagen formation pathways are still involved in maintaining the vascular lobular structure and influence the proliferation and involution of the lesions. By measuring the levels of laminin, FN1 and collagen, it may be possible to assess the progression of hemangiomas.

This study has several limitations. First and foremost, the vascular tumor sample from this patient was not additionally preserved. The sample was divided into two parts: one part was used by the hospital to perform postoperative pathological sectioning and immunohistochemical staining, which confirmed the diagnosis of mixed-type hemangioma; the other part was sent to a research institution for single-cell sequencing. Thus, there was no remaining sample available to validate the single-cell sequencing results, such as through qPCR, WB, immunofluorescence staining, or immunohistochemistry. Second, the single-cell data was derived from the tumor sample of a single patient, so the results lack generalizability. Given the rarity of intracardiac vascular tumors, we have not yet collected a second similar sample. Furthermore, the lack of normal control samples is another limitation, which has impacted our inferCNV analysis, enrichment analysis, and cell communication analysis. As a compensation, endothelial cell was divided into two populations based on postoperative immunohistochemical results, and Cluster 2, which does not express tumor cell-specific markers, served as the control group. In future studies, increasing the number of samples and obtaining normal control data will be of great importance.

## Conclusion

In summary, this scRNA-seq analysis of the intracardiac hemangioma has revealed transcriptional characteristics of various cell types of cardiac hemangioma, especially for endothelial cells. Endothelial cells involved in hemangioma formation exhibited functions primarily related in inflammation regulation, immune responses, and angiogenesis. A special subset of endothelial cells appears to be contributed significantly in vasomotor activities. Furthermore, according to cell communication analysis, we identified key regulatory roles of monocytes/macrophages and myofibroblasts in the formation process of cardiac hemangioma. The elevated expression of the VEGF pathway suggests strong angiogenic activity in endothelial cells. While the high expression of FN1 and collagen may be associated with the regression process of hemangioma. This study represents the first single-cell sequencing investigation of intracardiac hemangioma, providing novel insights and potential therapeutic targets for the treatment of this condition.

## Data Availability

The original contributions presented in the study are publicly available. This data can be found here: BioProject (PRJNA1365795, https://www.ncbi.nlm.nih.gov/bioproject/PRJNA1365795), BioSample (SAMN53293549, https://www.ncbi.nlm.nih.gov/biosample/SAMN53293549), and SRA (SRP646055, https://www.ncbi.nlm.nih.gov/sra/?term=SRP646055).
