# Supplementary material for: Case Report: Single-cell RNA sequencing reveals cellular and molecular mechanisms in newborn cardiac hemangioma formation
Source: Front Cardiovasc Med. 2025 Dec 8;12:1682677. doi: 10.3389/fcvm.2025.1682677 (PMC12722891; doi:10.3389/fcvm.2025.1682677)
Supplement: Supplementary file 1 [file Supplementaryfile1.docx]

**Supplementary Materials**





**Supplementary Figure S1. Single-cell RNA sequencing data quality control and cell clustering.** A & B, Scatter plots (A) and violin plots (B) show the conventional quality control indicators before quality control and normalization. C & D, Scatter plots (C) and violin plots (D) show the quality control indicators after quality control and normalization. E, Principal component analysis plot showing the distribution of cells based on the first two principal components. F, Elbow plot of the relationship between the number of principal components and the proportion of variance explained, used to determine the optimal number of principal components for downstream analysis. G, Cluster tree plot showing the hierarchical clustering counts and relationship at different resolution. H, Uniform Manifold Approximation and Projection plots of cells clustered at different resolutions (0.2, 0.5, 0.8, 1.0), with each color representing a distinct cell cluster.

Supplementary Figure S2


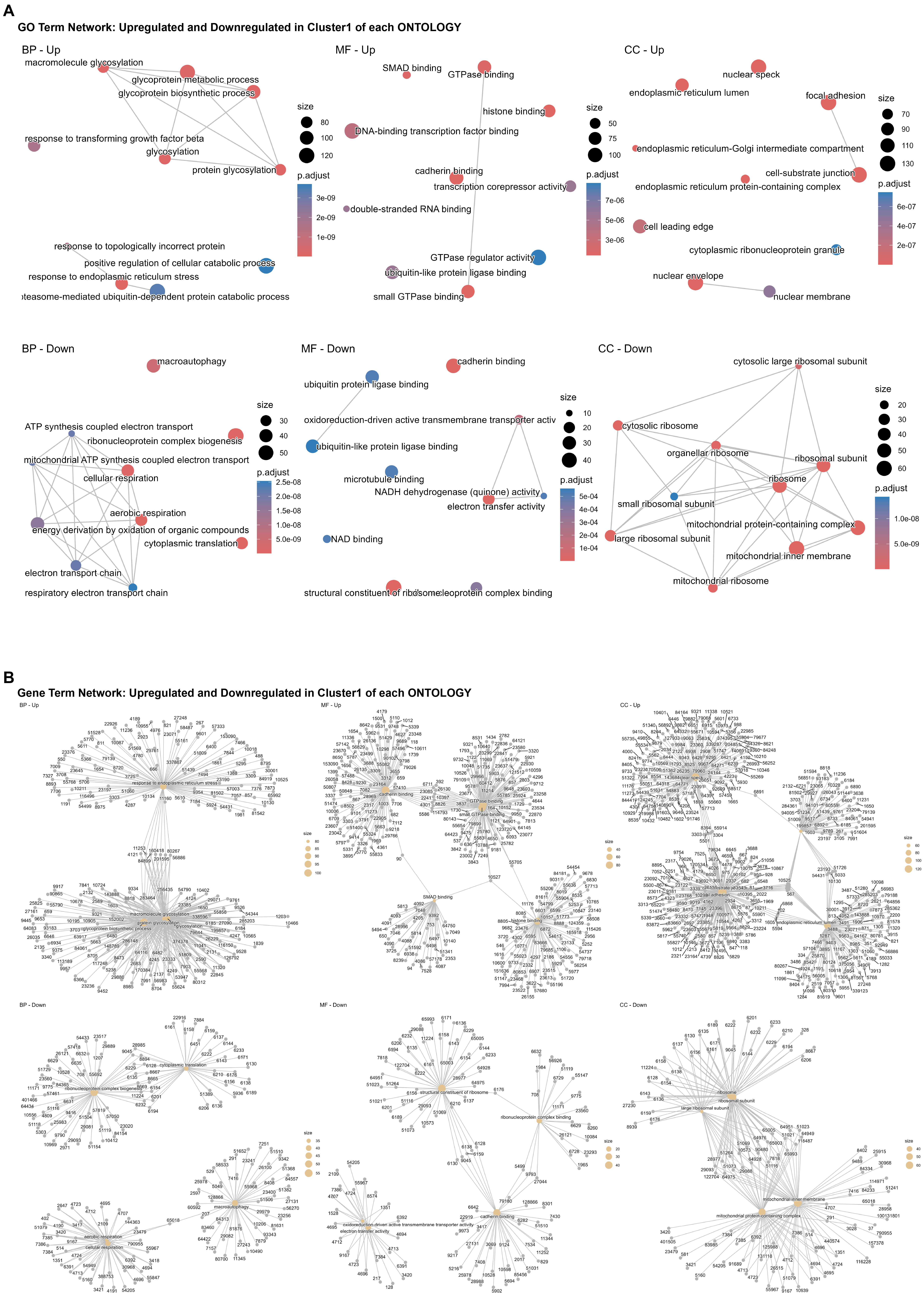


**Supplementary Figure S2. Detailed enrichment analysis network for endothelial cell clusters.** A & B, complete Gene Ontology term network (A) and Gene term network (B) analysis of Endothelial cell cluster 1, where upregulation and downregulation are obtained by comparison with Endothelial cell cluster 2.


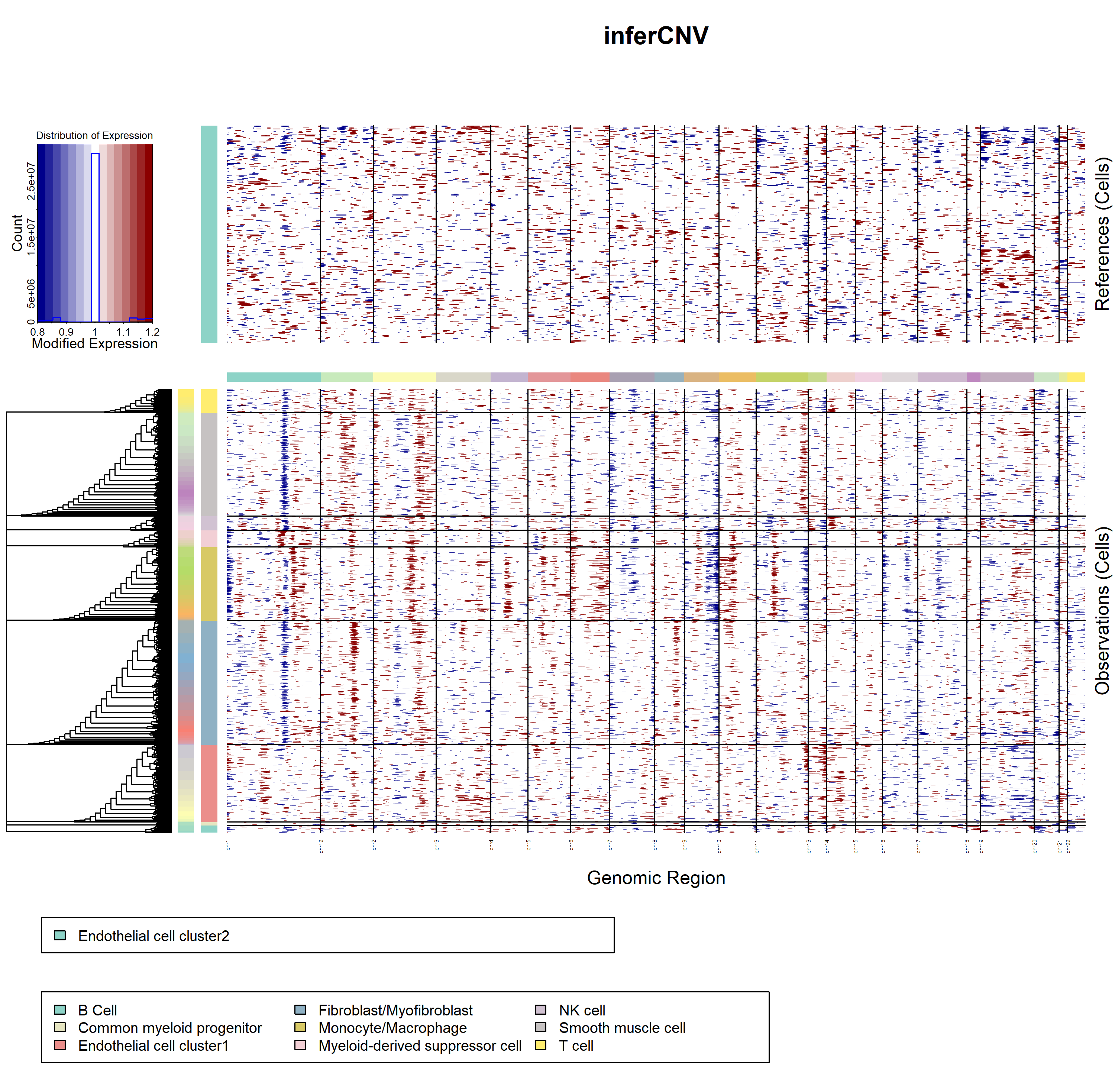


**Supplementary Figure S3. Single-cell copy-number variation analysis of hemangioma using inferCNV.** The horizontal axis represents genomic positions across chromosomes, included *chr1* to *chr22*. The vertical axis is partitioned into two segments: the upper segment depicts Reference cells (Endothelial cell cluster 2), while the lower segment depicts Observation cells.


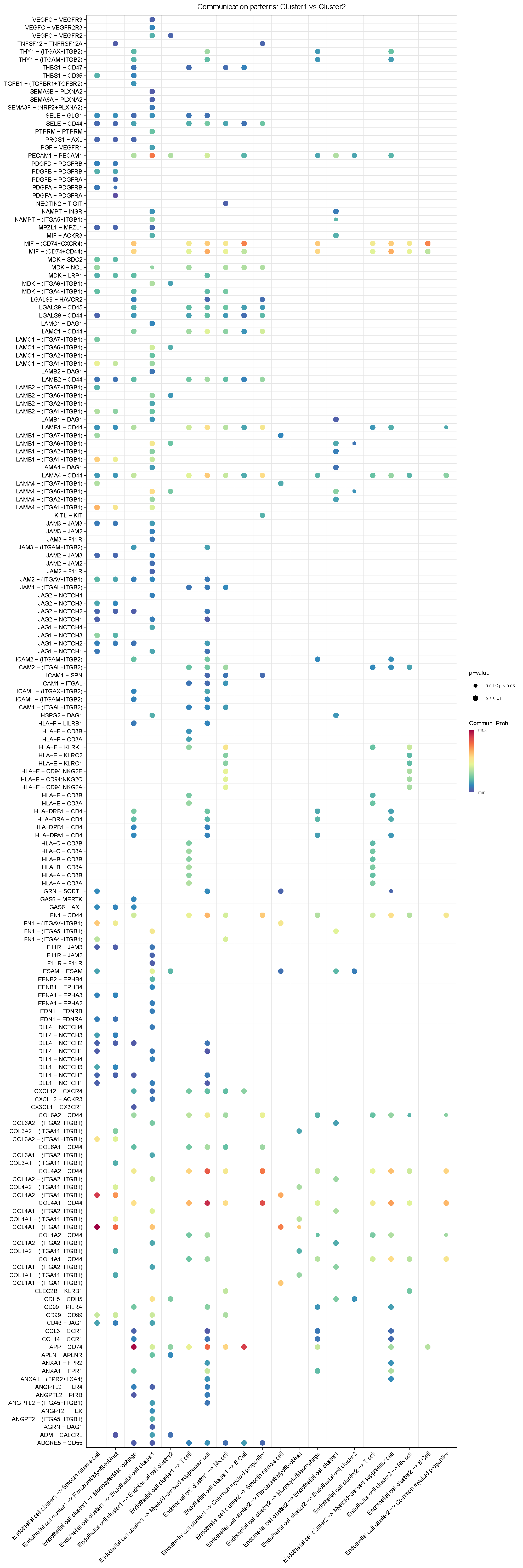


**Supplementary Figure S4. Detailed analysis of communication patterns in endothelial cell clusters.** Dot plots show the differences in various receptor-ligand cell communication patterns and their target cell types between Endothelial cell cluster 1 and Endothelial cell cluster 2.
